# Prosthetic joint infections: outcome after treatment in a case series

**DOI:** 10.1186/1471-2482-13-S1-A41

**Published:** 2013-09-16

**Authors:** Donato Rosa, Giovanni Balato, Tommaso Ascione, Giovanni Ciaramella, Sigismondo Di Donato, Vincenzo Crispino

**Affiliations:** 1 Department of Orthopaedic Surgery, Federico II University, Naples, Italy; 2 Department of Infectious Diseases, D. Cotugno Hospital, Naples, Italy

## Introduction

Prosthetic joint infections (PJI) are one of the most complication of joint replacement surgery and report a considerable disability and cost. This complication occurs in 0.8-1.9% of knee prostheses (TKA) and 0.3-1.7% of hip replacement (THA) [1]. These percentages may be underestimated because of the ability of some bacteria to grow in community of aggregation on the surface of the prosthesis, defined biofilm, which makes difficult their isolation by standard culture tests. In fact, in a significant percentage of prosthetic infections, the responsible agent remains unknown, and this may affect the outcome [2,3]. The classification of implant-associated infections is related to the onset of symptoms after implantation. Early infection is defined as appearance of the first signs and symptoms of infection during the first 3 months after surgery. However, some authors limit these surgical site infection to the first 4- weeks [4]. Delayed manifestation is defined as an infection in which the first signs and symptons appear between 3months and 2 years post-surgery, and late manifestation is defined as the appearance of first signs and symptons of infection > 2 years post- surgery. Each type has specific etiopathogenic properties that influence the therapeutic options. Outcome data on treatment are limited. Aim of this study was to evaluate the characteristics of patients with PJI and their outcome after treatment.

## Methods

In an observational study we included the cases of PJI referred to our Department during the last 3 years. The presence of at least one of the following findings confirm the infection [5]: I) two or more cultures of joint aspirates or cultures of intraoperative specimens yielding the same microorganism; II) purulence surrounding the prosthesis at the time of explantation; III) acute inflammation detected on histopathological examination of peroprosthetic issue; IV) a sinus tract that communicated with the prosthesis. Cure was defined by clinical, microbiologic and laboratory (normalization of erythrocyte sedimentation rate and C-reactive protein level) evidences and, as assessed 6 months after the end of therapy.

## Results

Twenty-two cases of PJI (median age 65 years), were observed (10 THA, 12 TKA). Co-morbidity were reported in 12 (54%) patients, diabetes mellitus, cardiovascular co-morbidities, and chronic liver disease were retrieved more frequently. Eight cases were observed within 3 months from surgical procedure (early infection), and 13 cases were observed after >3 months (delayed infections). Early infections ware caused by Staphylococcus aureus in six patients (2 methicillin resistant), in one patient we detected Pseudomonas aeruginosa and no microbiologic evidence was reported in one case. All early infections received debridement and retention of prosthesis followed by antibiotic therapy (success rate 75%). Delayed infections were caused by coagulase negative Staphylococci in 10 patients, in three patients no microbiologic evidence was reported. Delayed infections were treated with a two-stage exchange in 11 cases (success rate 85%). The remaining 2 cases received long-term suppressive antibiotic treatment without prosthetic joint replacement, because of refusal of further surgical procedure or because of co-morbidity.

## Conclusion

Prosthetic joint infections are frequently sustained by multi-drug resistant bacteria. Debridement followed by antibiotic treatment is a successful procedure for early infection. Satisfactory outcomes can be obtained also with two-stage revision. Patients with delayed infections have to be evaluated on the basis of underlying conditions to establish the surgical and antimicrobial approaches.
